# The role of explant type and selective agent application in the initial transformation rate of Lens culinaris Medik.

**DOI:** 10.18699/vjgb-26-26

**Published:** 2026-04

**Authors:** T.V. Dyubenko, K.V. Smirnov, V.E. Tvorogova

**Affiliations:** Federal Research Center the N.I. Vavilov All-Russian Institute of Plant Genetic Resources (VIR), St. Petersburg, Russia; Saint Petersburg State University, St. Petersburg, Russia All-Russia Research Institute for Agricultural Microbiology, Pushkin, St. Petersburg, Russia; Saint Petersburg State University, St. Petersburg, Russia Sirius University of Science and Technology, Sirius Federal Territory, Krasnodar region, Russia

**Keywords:** lentil, hygromycin, GUS, GFP, transformation, Lens culinaris, чечевица, гигромицин, GUS, GFP, трансформация, Lens culinaris

## Abstract

Lens culinaris Medik. (lentil) is an agronomically important leguminous species, but its genome modification is rarely used for obtaining new varieties, probably due to a low efficiency of transformation protocols. Development of universal, genotype-independent protocols for obtaining transgenic plants usually relies, among other factors, on the possibility of obtaining a substantial number of transgenic cells in vitro. This study aimed to adapt a previously developed Agrobacterium-mediated transformation protocol, used for a related legume, for the production of transgenic callus tissue in L. culinaris. We used two different markers of transgenic tissue, beta-glucuronidase and green fluorescent protein, to find an optimal type of explant for obtaining transgenic tissue in lentil. We also evaluated the impact of hygromycin, a common selective agent, on the amount of transgenic tissue in developing transformed explants of L. culinaris. According to our results, the transformation protocol commonly used for Medicago truncatula Gaertn. leaf explants can also be applied for obtaining transgenic calli from L. culinaris shoot apices. Explants from shoot apices demonstrated higher initial transformation rate in comparison with explants from roots, stems and leaves. Moreover, explants of different types, which were cultivated on medium without hygromycin, developed significantly fewer calli expressing reporter genes than those grown on hygromycin-containing medium, confirming that hygromycin may be used as an effective selection agent for lentil. During our analysis, we noticed GUS-like staining in calli which didn’t contain plasmids for GUS gene expression. This can be explained with so-called intrinsic GUS-like activity, which was described in previous research. These data can be used for further development of effective and universal L. culinaris transformation and genome editing protocols.

## Abstract (ru)

Lens culinaris Medik. (чечевица) – важная сельскохозяйственная культура из семейства бобовых, но модификация ее генома редко используется для создания новых сортов. Вероятно, это связано с низкой эффективностью существующих протоколов трансформации. Как правило, разработка универсальных, независимых от генотипа протоколов получения трансгенных растений зависит, среди прочего, от возможности образования существенного количества трансгенных клеток in vitro. В настоящем исследовании мы предприняли попытку адаптировать разработанный ранее для другого бобового протокол агробактериальной трансформации для получения трансгенной каллусной ткани у L. culinaris. Чтобы оценить эффективность трансформации, мы выбрали две независимые репортерные системы: бета-глюкуронидазу и зеленый флуоресцентный белок. С помощью этих систем мы нашли оптимальный тип экспланта для создания каллусной ткани чечевицы, экспрессирующей рекомбинантную ДНК. Мы также оценили влияние гигромицина, одного из распространенных селективных агентов, на количество трансгенной ткани в развивающихся трансформированных эксплантах L. culinaris. Согласно нашим результатам, протокол трансформации, который обычно применяется для эксплантов листьев Medicago truncatula Gaertn., может быть использован и для получения трансгенных каллусов из верхушек побегов L. culinaris. Экспланты из апексов побегов продемонстрировали более высокую первичную эффективность трансформации по сравнению с эксплантами из корней, стеблей и листьев. Кроме того, экспланты разных типов, культивируемые на среде без гигромицина, образовывали значительно меньше каллусов, экспрессирующих репортерные гены, по сравнению с эксплантами, выращиваемыми на среде с гигромицином. Это подтверждает возможность использования гигромицина в качестве эффективного селективного агента для чечевицы. В ходе нашего анализа мы также обнаружили GUS-подобное окрашивание в каллусах, не содержащих плазмид для экспрессии гена GUS, что можно объяснить так называемой внутренней GUS-подобной активностью, которая была описана в предыдущих исследованиях. Эти данные могут быть полезны для дальнейшей разработки эффективных и универсальных протоколов трансформации и редактирования генома L. culinaris

## Introduction

Lentils (Lens culinaris Medik.) are one of the oldest domesticated crops (Erskine et al., 2009), the cultivation history of which dates back over 8,000 years (Liber et al., 2021). Lentil production has doubled since the beginning of the 21st century (Kaale et al., 2023), coinciding with a rising societal interest in plant-based meat alternatives and growing trends towards more sustainable food production (Runte et al., 2024). Being a leguminous plant, L. culinaris is used both as a nitrogen-fixing crop improving the soil fertility and as a source of protein for human nutrition. The level of carbohydrates, including starch and dietary fiber, is also quite high in lentils, making them a valuable crop among other legumes. Furthermore, biochemical properties, specific for different lentil cultivars, provide a wide range of lentil applications in food production (Kaale et al., 2023).

Despite many features, which make lentils a valuable crop, its production and usage are related with diverse obstacles, including undesirable traits common for most legumes and also drawbacks which are specific for this culture. The features which require alleviation in lentil, include high content of different anti-nutrients, such as phytate, as well as low levels of some amino acids (Joshi et al., 2017), pod shattering (Cao et al., 2024), etc.

Advancements in next-generation sequencing led to the discovery of many genetic markers and genes related to different desirable and undesirable traits in lentils (Kumar et al., 2023). These data can be widely used for selection and breeding of lentil cultivars with specific useful features. However, currently this information may rarely be used for genome modification of lentils, because, similarly to many legumes, the transformation and regeneration effectiveness of this species is quite low. There are only a few studies on L. culinaris transformation, and, to our knowledge, all of them report on low effectiveness of obtaining transgenic lentil plants (Gulati et al., 2002; Celikkol Akcay et al., 2009; Khatib et al., 2011; Chopra et al., 2012; Das et al., 2012, 2019; Polowick, Yan, 2023), which doesn’t exceed 7 percents (i. e. 7 transgenic plants per 100 explants) for individual cultivars. Moreover, these protocols are developed for specific lentil varieties and, therefore, their effectiveness may depend greatly on genotype transformed.The transformation effectiveness depends both on the success of foreign DNA introduction into plant cells and on further fate of the transformed cell, i. e. its ability to give rise to a new plant. Consequently, these parameters are influenced by many factors accompanying transformation, including the method of transformation, explant type, cultivation medium and the method of transformant selection. A significant part of plant transformation protocols includes the stage of obtaining transgenic callus. At this stage, modified cells proliferate and some of them acquire the capacity for regeneration. Therefore, the efficiency of transgenic callus formation plays an important role in overall transformation efficiency. Moreover, maximization of transgenic tissue yield for a plant species facilitates the screening for morphogenic regulators, i. e. genes that are able to induce plant regeneration from transgenic tissue and can be used for effective cultivar-independent transformation (Bakulin et al., 2025).

In the current study, we made an attempt to find an optimal type of explant for obtaining transgenic callus tissue in lentil. We also evaluated the impact of hygromycin, a common selective agent, on the amount of transgenic tissue in developing transformed explants of L. culinaris. We built our transformation protocol on the highly effective transformation system used for Medicago truncatula, a well-studied model leguminous plant (Cosson et al., 2006). This system includes the formation of a significant amount of transgenic callus tissue. Moreover, as a model legume, M. truncatula provides an efficient platform for identifying key morphogenic regulators (Yakovleva et al., 2024), which can subsequently be validated in L. culinaris. The optimized protocol presented in this study provides a foundation for such validation experiments

## Materials and methods

In the current study, we made an attempt to find an optimal type of explant for obtaining transgenic callus tissue in lentil. We also evaluated the impact of hygromycin, a common selective agent, on the amount of transgenic tissue in developing transformed explants of L. culinaris. We built our transformation protocol on the highly effective transformation system used for Medicago truncatula, a well-studied model leguminous plant (Cosson et al., 2006). This system includes the formation of a significant amount of transgenic callus tissue. Moreover, as a model legume, M. truncatula provides an efficient platform for identifying key morphogenic regulators (Yakovleva et al., 2024), which can subsequently be validated in L. culinaris. The optimized protocol presented in this study provides a foundation for such validation experiments

Plasmids used in the study. For L. culinaris transformation, two plasmids were used: pMDC32_GUS (Tvorogova et al., 2019) with the beta-glucuronidase (GUS) gene and pMDC32_eGFPer obtained in this study. To construct the pMDC32_eGFPer plasmid, the eGFPer gene, encoding the enhanced green fluorescent protein (GFP) with endoplasmic reticulum localization signal, was amplified from the pB7WG2D vector (Karimi et al., 2002) with primers containing attB1 and attB2 sites (forward primer: GGGGACAAGTTTGT ACAAAAAAGCAGGCTTCATGGTGAAGACTAATC TTTTTCTC; reverse primer: GGGGACCACTTTGTACA AGAAAGCTGGGTCTTACAGCTCGTCCTTCTTG) and cloned into the pDONR207 entry vector (Invitrogen) and then to the pMDC32 vector (Curtis, Grossniklaus, 2003) using Gateway technology (Invitrogen).

Agrobacterium-mediated transformation. For explants preparation, seeds of L. culinaris were incubated in concentrated sulfuric acid for 7 minutes, rinsed with tap water 7 times, then incubated in 5–7 % sodium hypochlorite solution (the “Belizna” bleach) for 8 minutes and rinsed with sterile water 10 times in a laminar hood. After sterilization, seeds were put on modified Fahraeus medium (Fåhraeus, 1957; Tvorogova et al., 2019) and left to germinate for 12 days at 21–24 °C, 16 h (light)/8 h (dark) photoperiod. After that, germinated seedlings were used to prepare explants. Root explants (about 10–15 mm length) were obtained from root tips, shoot apex explants (about 10–15 mm length) were taken from the main and/ or lateral shoots. Stem explants (about 3–8 mm length) were obtained from stem fragments between the first and the second leaves of the seedlings. For leaf explants, one or two first true leaves were taken. Seedling growth was asynchronous, and the explant size depended on the seedling development stage.

For Agrobacterium-mediated transformation itself, a modified protocol of Cosson et al. (2006) for M. truncatula Gaertn. was used. Agrobacterial strain carrying either pMDC32_GUS or pMDC32_eGFPer plasmids was recovered from glycerol stock (500 μl of 50 % glycerol mixed with 500 μl of overnight stationary-phase culture) stored at –80 °C and cultured on solid YEP medium (Table S1)1 containing kanamycin (50 mg/l) and rifampicin (40 mg/l). Kanamycin resistance was provided by the pMDC32_GUS or pMDC32_eGFPer plasmid, whereas rifampicin resistance was characteristic of the AGL1 strain. On such medium, bacteria were cultivated at 30 °C for 1–2 days. Then, a small amount of bacteria from the Petri dish was sown to the liquid YEP with the same concentrations of antibiotics, in which they were grown overnight in a thermoshaker at 30 °C and 200 rpm. On the next day, 2 ml of bacterial culture were transferred to 50 ml of AB-MES medium (Wu et al., 2014, Table S1) with the addition of 40 mg/l (200 μM) acetosyringone, kanamycin (50 mg/l) and rifampicin (40 mg/l). Bacteria were cultivated in this medium at 30 °C and 200 rpm for about 3 hours. Then, they were centrifuged (4,000g, 15 minutes, room temperature) and resuspended in liquid ABM-SH medium (Table S1) with 4 mg/l (18 μM) 2,4-dichlorophenoxyacetic acid (2,4-D), 0.5 mg/l (2.22 μM) 6-benzylaminopurine (BAP), and 40 mg/l (200 μM) acetosyringone to the final OD600 0.1. ABM-SH represented a mixture of 0.5X AB-MES components and 0.5X modified liquid PCI-4 medium (Hoffmann et al., 1997) components, similarly to the infiltration medium from the AGROBEST protocol for Arabidopsis thaliana (L.) Heynh. (Wu et al., 2014).

Supplementary Materials are available in the online version of the paper: https://vavilovj-icg.ru/download/pict-2026-30/appx17.pdf


Prepared explants were incubated in Agrobacterium suspension in ABM-SH medium for 15 minutes with slow gentle agitation. After the inoculation stage, explants were blotted on sterile filter paper, then put on solid ABM-SH medium (Table S1) and cultivated in the dark at 21–24 °C for about 36 hours. After cocultivation, explants were put on solid PCI-4 medium (Hoffmann et al., 1997, Table S1) for callus formation with 4 mg/l (18 μM) 2,4-D, 0.5 mg/l (2.22 μM) BAP, and with the addition of cefotaxime (250 mg/l), to eliminate agrobacteria. In some experiments, hygromycin (10 mg/l), necessary for the selection of transgenic plant cells, was added to the medium. On this medium, explants were cultivated for 44 days, with transfer on the fresh medium every 14–16 days. At the end of cultivation, explants were either stained with X-Gluc and analysed with stereomicroscope to detect GUS expression, or analysed with the fluorescent stereomicroscope to detect GFP fluorescence

GUS staining. For GUS staining, X-Gluc substrate (Sisco Research Laboratories Pvt. Ltd.) was used. Plant tissues were collected in NT buffer (100 mM Tris-HCl, 50 mM NaCl) and then transferred to X-Gluc buffer (100 mM Tris-HCl, 50 mM NaCl, 0.2 mM K3[Fe(CN)6], 2.5 mM X-Gluc), where they were incubated at 37 °C overnight. After staining, tissues were transferred to 70 % ethanol. Photos of single explants were taken with a ZEISS SteREO Discovery.V12 stereomicroscope. Photos of Petri dishes with explants were taken with the Vilber Fusion-FX6.Edge imaging system. For microscopic images, pieces of callus tissue were squashed and examined under a Leica DM500 microscope.

Statistical analysis and software. For evaluation of explant type and hygromycin presence impact, 20–28 explants per group were used. Statistical significance of differences was evaluated with Fisher’s exact test with Bonferroni adjustment for multiple comparisons, if necessary. Statistical analysis of results and diagram drawing were performed in the R studio using the vcd (Meyer et al., 2006, 2024) and dplyr (Wickham et al., 2025) packages. Final figures were created using the Inkscape software.

## Results

**Evaluation of transgenic tissue development from different explant types using the GFP marker**

We decided to build our transformation protocol on the transformation system used for M. truncatula (Cosson et al., 2006), in which transgenic plants are developed from transformed leaf explants through somatic embryogenesis. To find the optimal variant of explant type for transformation, we performed Agrobacterium-mediated transformation of root, stem, leaf and shoot apex explants with plasmid pMDC32_eGFPer for eGFPer overexpression. After cocultivation, explants were transferred to the callus-inducing medium with or without hygromycin. The concentrations of plant growth regulators and cefotaxime for agrobacteria elimination were identical to the ones used for M. truncatula, but the concentration of hygromycin was lower (10 mg/l instead of 25 mg/l used for M. truncatula), because 10 mg/l concentration is successfully used for other legumes to obtain transgenic tissue (Olhoft et al., 2003; Li et al., 2023; Tisseyre et al., 2024). After 46 days of cultivation on this callus-inducing medium, calli were developed from all types of explants both on the medium with hygromycin and hygromycin-free medium (Figs. S1, S2). We analysed GFP fluorescence to evaluate the effectiveness of transformation.

**Fig. 1.**
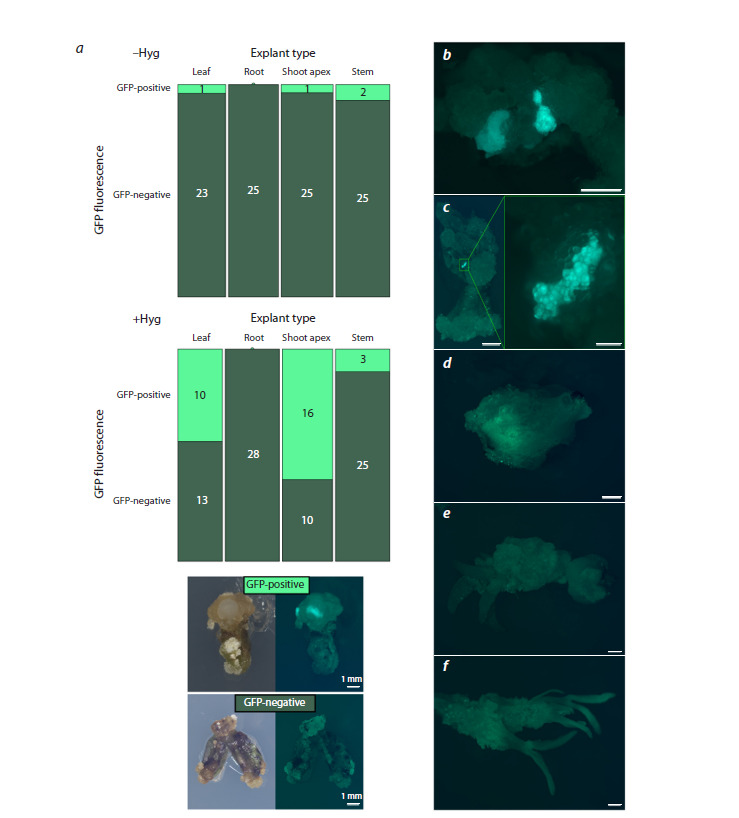
a, Mosaic plots demonstrating the number of calli with GFP fluorescence (GFP-positive) and without GFP fluorescence (GFP-negative) developed from different types of explants, on the medium without hygromycin (above) or on the medium with addition of 10 mg/l hygromycin (below). In a legend below, the photos of explants were taken in white light (left) and fluorescent (right) light. b–d, Calli with GFP fluorescence. e, f, Calli without detectable GFP fluorescence, obtained after explant transformation with the pMDC32_GFP (e) or pMDC32_GUS (f ) plasmids. Size bar is 1 mm for b–f and 100 μm for the inset in c.

To avoid false positive results, we also analysed the green fluorescence in the explants transformed with pMDC32_GUS construction, which didn’t contain the GFP gene. Bright GFP fluorescence in separate tissue areas (Fig. 1b, c), as well as less bright diffuse fluorescence (Fig. 1d ), were easily distinguishable from autofluorescence (Fig. 1e, f ), which was observed in control pMDC32_GUS explants. It is worth noting that some fluorescent areas with weak signal, as well as autofluorescent areas, can only be seen with high magnification (60× and more). Therefore, for robust comparative analysis, it was crucial to evaluate the presence of fluorescence for all explants without changing magnification and other visualization settings on the stereomicroscope

According to our results, on the medium supplemented with 10 mg/l hygromycin, the type of explant had an impact on the frequency of fluorescent calli (Fisher’s exact test, p- value 4.359e–08). The highest frequency of transformed calli with GFP fluorescence was detected for explants from shoot apices (Fig. 1a). Leaf explants also demonstrated a rather high frequency of transformation, whereas only 3 out of 28 stem explants developed fluorescent calli. Calli from the root explants didn’t demonstrate any transgenic fluorescent tissue at all (Fig. 1a). Overall, shoot apex explants formed significantly more fluorescent calli in comparison with explants from roots or stems (Fisher’s exact test with Bonferroni adjustment for multiple comparisons, p-values 1.51e–06 and 8.46e–04, respectively), whereas differences between explants from shoot apices and leaves were not statistically significant.

At the same time, explants of all types, cultivated on medium without hygromycin, developed significantly fewer fluorescent calli than those grown on hygromycin-containing medium (Fisher’s exact test, p-value 2.09e–06), confirming that hygromycin may be used as an effective selection agent for lentil (Fig. 1a).

**Evaluation of transgenic tissue development from different explant types using GUS marker**

**Fig. 2.**
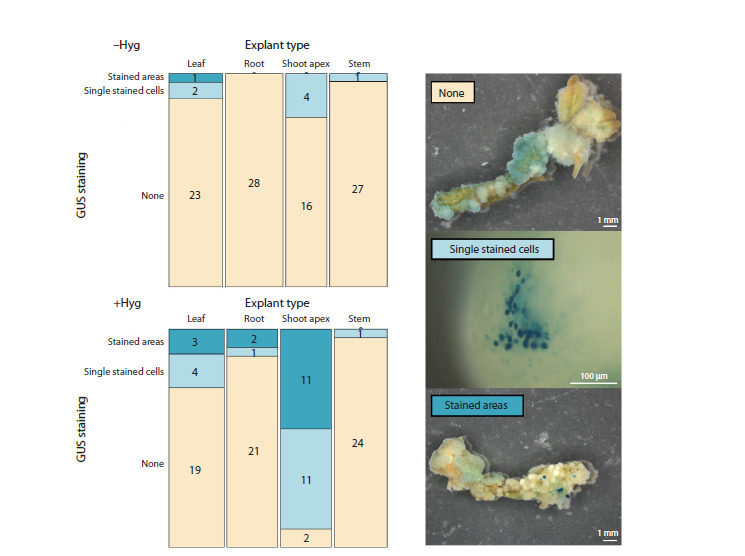
Mosaic plots demonstrating the number of stained and non-stained calli developed from different types of explants after transformation with pMDC32_GUS plasmid, on the medium without hygromycin (above) or on the medium with addition of 10 mg/l hygromycin (below). The legend on the right demonstrates calli belonging to different categories relative to GUS staining: none – no detectable GUS staining apart from the light-blue color characteristic of endogenous GUS-like activity; single stained cells – small spots of GUS staining visible only with high (about 80×) magnification, stained areas – patches of GUS staining visible with naked eye or with low magnification.

We performed a similar experiment with transformation of different explant types using GUS, another transgenic tissue marker. Explants were transformed with the pMDC32_GUS plasmid (Tvorogova et al., 2019) using the protocol mentioned above. After 46 days of cultivation (starting from the day of transformation), GUS activity was evaluated in explants. Usage of GUS allowed us to detect single stained cells (at about 80× magnification on stereomicroscope), which we were not able to visualize with the GFP marker. The results of transgenic tissue detection were similar to those obtained using the GFP marker. On the media with hygromycin, the highest transformation efficiency was shown for shoot apex explants, followed by leaf explants. Both root and stem explants demonstrated a low transformation rate (Fig. 2). In total, shoot apex explants formed significantly more stained calli in comparison with explants from leaves, roots or stems (Fisher’s exact test with Bonferroni adjustment for multiple comparisons p-values 6.243734e–05, 2.191140e–07 and 7.429123e–10, respectively). In contrast, all types of explants grown on the hygromycin-free media mostly didn’t develop detectable transgenic tissues. The difference was statistically significant when compared to explants cultivated on hygromycin-containing medium (Fisher’s exact test, p-value 5.842e–12), confirming our results obtained for the GFP marker (Fig. 1).

**Evaluation of GUS false positive staining**

**Fig. 3.**
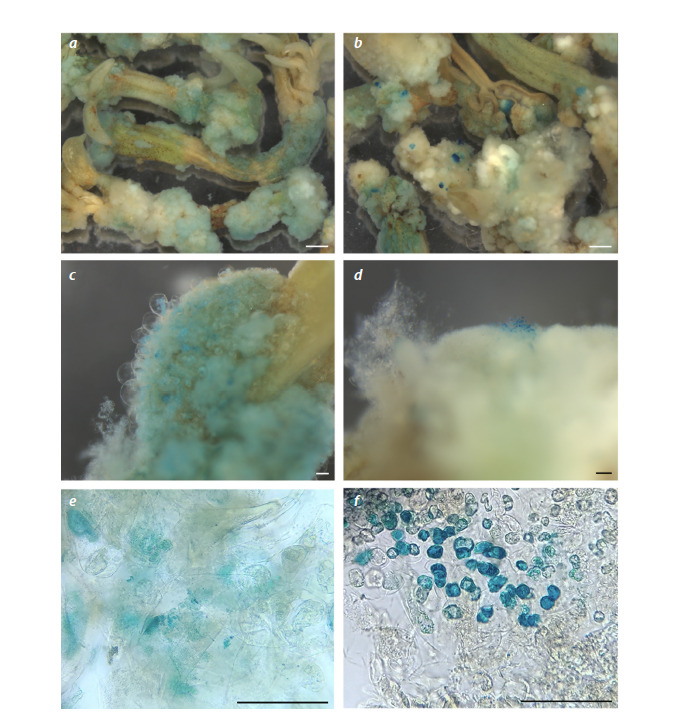
GUS staining of control calli obtained from explants transformed with the pMDC32_eGFPer plasmid (a, c, e) or calli obtained from explants transformed with the pMDC32_GUS plasmid (b, d, f ). Size bar is 1 mm for a, b and 100 μm for c–f.

During the experiment with GUS activity analysis described above, we also performed GUS staining of explants transformed with the pMDC32_eGFPer construction, which didn’t carry the GUS gene. Unexpectedly, these control explants also demonstrated blue staining, although the staining pattern differed from the one provided by true GUS expression (Hu et al., 1990). In false positives, the blue coloration was more diffuse and didn’t have distinct borders compared with true GUS staining (Fig. 3).

Microscopic analysis also revealed differences between false positive and true GUS staining. GUS-expressing cells had deep blue coloring (Fig. 3f ), which was not the case for false positive GUS-like staining (Fig. 3e). Therefore, the GUS marker can be used to detect transgenic tissue in lentil, but the staining of negative controls is necessary

## Discussion

Transformation is a powerful tool for obtaining new varieties of different plant species (Garcia Ruiz et al., 2018; Noack et al., 2024), which is why improving its effectiveness for lentil is important. Nevertheless, current L. culinaris transformation protocols have rather low efficiency, probably due to the low regeneration rate. One of the possible solutions for this problem is the usage of morphogenic regulators, i. e. genes that can be added to transformation vectors and that are able to induce proliferation of transgenic cells and plant regeneration from transgenic tissue. Still, the search for such morphogenic regulators, suitable for lentils, can be hampered with low initial transformation rate, i. e. low effectiveness of transgenic callus formation. Therefore, the development of effective protocols for maximizing the transgenic tissue amount can be very useful for further optimization of obtaining transformed and edited plants

The protocols for obtaining transgenic lentil plants usually involve cotyledons and/or cotyledonary nodes, parts of embryos or embryo axes (Gulati et al., 2002; Cellikol Akcay et al., 2009; Khatib et al., 2011; Das et al., 2012, 2019; Polowick, Yan, 2023) or whole seeds (Chopra et al., 2012). In our study, we tried to perform Agrobacterium-mediated transformation using different types of explants obtained from 12-day old seedlings. According to our results, the transformation protocol, developed for M. truncatula leaf explants, can also be used for obtaining transgenic tissue from L. culinaris explants. Still, although the leaf explants are used in this protocol for M. truncatula, a different type of explant, shoot apex, gave better results for L. culinaris. It can possibly be related to a relatively high proportion of stem cells and, in general, proliferating cells in the shoot apex in comparison with other explant types. It would be interesting to evaluate the efficiency of usage of seedling shoot apices for M. truncatula transformation. The usage of hygromycin at the concentration 10 mg/l allowed us to obtain a significant amount of transgenic tissue from shoot apex explants. Nevertheless, the optimal hygromycin concentration has to be defined in the future.

There are also several studies aiming to obtain transgenic tissues in lentil from diverse explant types, including different parts of germinated seedlings (Warkentin, McHughen, 1992), cotyledonary nodes (Mahmoudian et al., 2002) and embryo axes (Lurquin et al., 1998), although in most cases, no selection agent and no 2,4-D-mediated callus induction was applied, in contrast to our research. As far as we know, the current study is also the first in which optimization of lentil transformation was performed with the usage of two different transgenic tissue markers

In a study by Warkentin and McHughen (1992) Agrobacterium- mediated transformation of lentil shoot apex explants yielded a high percentage of explants, which contained transgenic tissue, without selection for transgenic cells at early stages of cultivation. In our study, cultivation on the medium without a selection agent was not effective, whereas hygromycin application made it possible to obtain a significant amount of transgenic tissue. The discrepancy between our results and results obtained previously may be due to differences in genotypes, in vitro cultivation and transformation conditions.

In experiments on evaluation of in vitro regeneration capacity in lentil, explants from shoot tips were shown to give callus on the medium with 2,4-D, as well as regenerating shoots on other types of media, with the addition of BAP, IAA or NAA (Polanco et al., 1988). Shoot regeneration from shoot apex explants was also observed after Agrobacterium-mediated transformation, although shoots regenerated during kanamycin selection were non-transgenic (Warkentin, McHughen, 1992). Therefore, the development of a protocol for producing transgenic lentil plants from shoot tip explants may be feasible upon identification of suitable selection and regeneration conditions. Based on our data, hygromycin selection appears to be a promising approach.

During our analysis, we noticed the GUS-like staining in calli that didn’t contain plasmids for GUS gene expression. This can be explained with so-called intrinsic GUS-like activity, which was described in previous research (Hu et al., 1990). Indeed, beta-glucuronidase (GUS) genes or proteins were described in several species, including Scutellaria baicalensis (Zuo et al., 2025) and Zea mays (Muhitch, 1998), whereas endogenous GUS activity was detected in Brassica napus (Abdollahi et al., 2011), Oryza sativa, A. thaliana, Nicotiana tabacum and other plants (Sudan et al., 2006). Still, in other studies involving GUS staining of lentil explants, no false positive GUS staining was detected on control explants, which may be due to different staining buffer composition or different tissue features (Warkentin, McHughen, 1992; Lurquin et al., 1998; Mahmoudian et al., 2002).

## Conclusion

Our results show that Agrobacterium-mediated transformation of lentil shoot apices according to the protocol developed for M. truncatula, can be successfully used for obtaining transgenic callus tissues. These data can be used for further development of effective and universal lentil transformation and editing protocols.

## Conflict of interest

The authors declare no conflict of interest.

## References

Abdollahi M.R., Memari H.R., van Wijnen A.J. Factor affecting the endogenous
β-glucuronidase activity in rapeseed haploid cells: how to
avoid interference with the GUS transgene in transformation studies.
Gene. 2011;487(1):96-102. doi 10.1016/j.gene.2011.07.007

Bakulin S.D., Monakhos S.G., Bruskin S.A. Morphogenetic factors
as a tool for enhancing plant regeneration capacity during in vitro
transformation. Int J Mol Sci. 2025;26(17):8583. doi 10.3390/ijms
26178583

Cao Z., Socquet-Juglard D., Daba K., Vandenberg A., Bett K.E. Understanding
genome structure facilitates the use of wild lentil germplasm
for breeding: a case study with shattering loci. Plant Genome.
2024;17(2):e20455. doi 10.1002/tpg2.20455

Celikkol Akcay U., Mahmoudian M., Kamci H., Yucel M., Oktem H.A.
Agrobacterium tumefaciens-mediated genetic transformation of a
recalcitrant grain legume, lentil (Lens culinaris Medik). Plant Cell
Rep. 2009;28(3):407-417. doi 10.1007/s00299-008-0652-4

Chopra R., Aparna, Saini R. Use of sonication and vacuum infiltration
for Agrobacterium-mediated transformation of an Indian lentil
(Lens culinaris Medik.) cultivar. Sci Hortic. 2012;143:127-134. doi
10.1016/j.scienta.2012.06.019

Cosson V., Durand P., d’Erfurth I., Kondorosi A., Ratet P. Medicago
truncatula transformation using leaf explants. Methods Mol Biol.
2006;343:115-127. doi 10.1385/1-59745-130-4:115

Curtis M.D., Grossniklaus U. A gateway cloning vector set for highthroughput
functional analysis of genes in planta. Plant Physiol.
2003;133(2):462-469. doi 10.1104/pp.103.027979

Das S.K., Shethi K.J., Hoque M.I., Sarker R.H. Agrobacterium-mediated
genetic transformation in lentil (Lens culinaris Medik.) followed
by in vitro flowering and seed formation. Plant Tissue Cult Biotechnol.
2012;22(1):13-26. doi 10.3329/ptcb.v22i1.11243

Das S.K., Shethi K.J., Hoque M.I., Sarker R.H. Agrobacterium-mediated
genetic transformation of lentil (Lens culinaris Medik.) with
chitinase
gene followed by in vitro flower and pod formation. Plant
Tissue Cult Biotechnol. 2019;29(1):99-109. doi 10.3329/ptcb.v29i1.
41982

Erskine W., Muehlbauer F., Sarker A., Sharma B. (Eds) The Lentil:
Botany, Production and Uses. Wallingford: CABI, 2009

Fåhraeus G. The infection of clover root hairs by nodule bacteria
studied by a simple glass slide technique. J Gen Microbiol. 1957;
16(2):374-381. doi 10.1099/00221287-16-2-374

Garcia Ruiz M.T., Knapp A.N., Garcia-Ruiz H. Profile of genetically
modified plants authorized in Mexico. GM Crops Food. 2018;9(3):
152-168. doi 10.1080/21645698.2018.1507601

Gulati A., Schryer P., McHughen A. Production of fertile transgenic
lentil (Lens culinaris Medik) plants using particle bombardment.
In Vitro Cell Dev Biol Plant. 2002;38(4):316-324. doi 10.1079/IVP
2002303

Hoffmann B., Trinh T.H., Leung J., Kondorosi A., Kondorosi E. A new
Medicago truncatula line with superior in vitro regeneration, transformation,
and symbiotic properties isolated through cell culture
selection. Mol Plant-Microbe Interact. 1997;10(3):307-315. doi
10.1094/MPMI.1997.10.3.307

Hu C.Y., Chee P.P., Chesney R.H., Zhou J.H., Miller P.D., O’Brien W.T.
Intrinsic GUS-like activities in seed plants. Plant Cell Rep. 1990;
9(1):1-5. doi 10.1007/BF00232123Joshi M., Timilsena Y., Adhikari B. Global production, processing and
utilization of lentil: a review. J Integr Agric. 2017;16(12):2898-
2913. doi 10.1016/S2095-3119(17)61793-3

Kaale L.D., Siddiq M., Hooper S. Lentil (Lens culinaris Medik) as nutrient-
rich and versatile food legume: a review. Legume Sci. 2023;
5(2):e169. doi 10.1002/leg3.169

Karimi M., Inzé D., Depicker A. GATEWAY™ vectors for Agrobacterium-
mediated plant transformation. Trends Plant Sci. 2002;7(5):
193-195. doi 10.1016/S1360-1385(02)02251-3

Khatib F., Makris A., Yamaguchi-Shinozaki K., Kumar S., Sarker A.,
Erskine W., Baum M. Expression of the DREB1A gene in lentil (Lens
culinaris Medik. subsp. culinaris) transformed with the Agrobacterium
system. Crop Pasture Sci. 2011;62(6):488-495. doi 10.1071/
CP10351

Kumar J., Gela T.S., Gupta D.S., Chandra A., Khazaei H. Recent advances
in lentil genetics, genomics, and molecular breeding. In:
Lentils: Production, Processing Technologies, Products, and Nutritional
Profile. Hoboken: John Wiley & Sons Ltd., 2023;25-43. doi
10.1002/9781119866923.ch2

Li G., Liu R., Xu R., Varshney R.K., Ding H., Li M., Yan X., … Luo Y.,
Gao S., Wei P., Zong X., Yang T. Development of an Agrobacteriummediated
CRISPR/Cas9 system in pea (Pisum sativum L.). Crop J.
2023;11(1):132-139. doi 10.1016/j.cj.2022.04.011

Liber M., Duarte I., Maia A.T., Oliveira H.R. The history of lentil
(Lens culinaris subsp. culinaris) domestication and spread as revealed
by genotyping-by-sequencing of wild and landrace accessions.
Front Plant Sci. 2021;12:628439. doi 10.3389/fpls.2021.
628439Lurquin P.F., Cai Z., Stiff C.M., Fuerst E.P. Half-embryo cocultivation
technique for estimating the susceptibility of pea (Pisum sati-vum
L.) and lentil (Lens culinaris Medik.) cultivars to Agrobacterium
tumefaciens. Mol Biotechnol. 1998;9(2):175-179. doi 10.1007/
BF02760819

Mahmoudian M., Yücel M., Öktem H.A. Transformation of lentil (Lens
culinaris M.) cotyledonary nodes by vacuum infiltration of Agrobacterium
tumefaciens. Plant Mol Biol Rep. 2002;20(3):251-257.
doi 10.1007/BF02782460

Meyer D., Zeileis A., Hornik K. The strucplot framework: visualizing
multi-way contingency tables with vcd. J Statistical Software. 2006;
17(3):1-48. doi 10.18637/jss.v017.i03

Meyer D., Zeileis A., Hornik K., Friendly M. vcd: visualizing categorical
data. R package version 1.4-12. 2024. URL: https://CRAN.Rproject.
org/package=vcd

Muhitch M.J. Characterization of pedicel β-glucuronidase activity in
developing maize (Zea mays) kernels. Physiol Plant. 1998;104(3):
423-430. doi 10.1034/j.1399-3054.1998.1040318.xNoack F., Engist D., Gantois J., Gaur V., Hyjazie B.F., Larsen A.,
M’Gonigle L.K., Missirian A., Qaim M., Sargent R.D., Souza-Rodrigues
E., Kremen C. Environmental impacts of genetically modified
crops. Science. 2024;385(6712):eado9340. doi 10.1126/science.
ado9340

Olhoft P.M., Flagel L.E., Donovan C.M., Somers D.A. Efficient soybean
transformation using hygromycin B selection in the cotyledonary-
node method. Planta. 2003;216(5):723-735. doi 10.1007/
s00425-002-0922-2

Polanco M.C., Peláez M.I., Ruiz M.L. Factors affecting callus and shoot
formation from in vitro cultures of Lens culinaris Medik. Plant Cell
Tissue Organ Cult. 1988;15(2):175-182. doi 10.1007/BF00035759

Polowick P.L., Yan W. A protocol for Agrobacterium-mediated genetic
transformation of Lens culinaris Medik (lentil). Plant Cell Tissue
Organ Cult. 2023;152(3):605-618. doi 10.1007/s11240-022-02434-x

Potsenkovskaia E., Tvorogova V., Yakovleva D., Zlydneva N., Lutova
L. Novel NF-Y genes expressed during somatic embryogenesis
in Medicago truncatula. Plant Gene. 2022;31:100364. doi 10.1016/
j.plgene.2022.100364

Runte M., Guth J.N., Ammann J. Consumers’ perception of plant-based
alternatives and changes over time. A linguistic analysis across three
countries and ten years. Food Qual Preference. 2024;113:105057.
doi 10.1016/j.foodqual.2023.105057

Sudan C., Prakash S., Bhomkar P., Jain S., Bhalla-Sarin N. Ubiquitous
presence of β-glucuronidase (GUS) in plants and its regulation
in some model plants. Planta. 2006;224(4):853-864. doi 10.1007/
s00425-006-0276-2

Tisseyre P., Cartieaux F., Chabrillange N., Gully D., Hocher V., Svistoonoff
S., Gherbi H. Setting up Agrobacterium tumefaciens-mediated
transformation of the tropical legume Aeschynomene evenia, a
powerful tool for studying gene function in nod factor-independent
symbiosis. PLoS One. 2024;19(4):e0297547. doi 10.1371/journal.
pone.0297547

Tvorogova V.E., Fedorova Y.A., Potsenkovskaya E.A., Kudriashov A.A.,
Efremova E.P., Kvitkovskaya V.A., Wolabu T.W., Zhang F., Tadege
M., Lutova L.A. The WUSCHEL-related homeobox transcription
factor MtWOX9-1 stimulates somatic embryogenesis in
Medicago truncatula. Plant Cell Tissue Organ Cult. 2019;138(3):
517-527. doi 10.1007/s11240-019-01648-w

Warkentin T.D., McHughen A. Agrobacterium tumefaciens-mediated
beta-glucuronidase (GUS) gene expression in lentil (Lens culinaris
Medik.) tissues. Plant Cell Rep. 1992;11(5-6):274-278. doi 10.1007/
BF00235081

Wickham H., François R., Henry L., Müller K., Vaughan D. dplyr:
a grammar of data manipulation. R package version 1.1.4. 2025.
URL: https://CRAN.R-project.org/package=dplyr

Wu H.-Y., Liu K.-H., Wang Y.-C., Wu J.-F., Chiu W.-L., Chen C.-Y.,
Wu S.-H., Sheen J., Lai E.-M. AGROBEST: an efficient Agrobacterium-
mediated transient expression method for versatile gene
function analyses in Arabidopsis seedlings. Plant Methods. 2014;
10(1):19. doi 10.1186/1746-4811-10-19

Yakovleva D., Efremova E., Smirnov K., Simonova V., Konstantinov
Z., Tvorogova V., Lutova L. The WOX genes from the intermediate
clade: influence on the somatic embryogenesis in Medicago
truncatula. Plants. 2024;13(2):223. doi 10.3390/plants13020223

Zuo X., Li P., Ren G., Bai Z., Jiang D., Liu C. Functional characterization
of β-glucuronidase genes involved in baicalein biosynthesis
from Scutellaria baicalensis based on transcriptome analysis. Int J
Mol Sci. 2025;26(5):4410. doi 10.3390/ijms26051793

